# Editorial: Advances in spinal muscular atrophy

**DOI:** 10.3389/fncel.2023.1178422

**Published:** 2023-03-17

**Authors:** Jianli Sun

**Affiliations:** Delaware Center for Neuroscience Research, Department of Biological Sciences, Delaware State University, Dover, DE, United States

**Keywords:** spinal muscular atrophy, survival motor neuron protein (SMN), pathology, multi-system, SMN-restoring therapies

Spinal muscular atrophy (SMA) is an autosomal recessive motor neuron disorder with an estimated incidence of 1 in 6,000 to 10,000 in live births, and a carrier frequency of 1/40 to 1/60 (Verhaart et al., 2017). Untreated SMA is the leading inherited cause of infant mortality. This disease is characterized by predominate proximal muscle weakness and atrophy. The SMA field has advanced remarkably since the discovery of the causative *survival motor neuron 1* (*SMN1*) gene in 1995 in SMA patients (Lefebvre et al., 1995). However, the mystery of how lower levels of the ubiquitous SMN protein cause the selective motor neuron (MN) degeneration in SMA is still unclear.

SMN is part of a large macromolecular complex consisting of SMN, Gemin 2–8, and Unrip (Pellizzoni et al., 2002). The core member of SMN complex, Gemin3 regulates MN survival through the NF-κB pathway (Arumugam et al., 2018). Miralles et al. in this Frontiers Research Topic presents evidence for an interdependent expression of SMN and Gemin3. They found that Gemin3 knockdown reduced protein expression levels of SMN and NF-κB pathway members, and caused significant neurite degeneration. Interestingly, SMN over-expression did not prevent neurite degeneration caused by the reduction of endogenous Gemin3, which suggested that Germin3 may be involved in cell degeneration independent of SMN.

Accumulating evidence suggests the activation of the tumor suppressor p53 pathway may be a major contributor to MN degeneration in SMA (Murray et al., 2015; Nichterwitz et al., 2020). However, it is not clear which p53 downstream effectors are involved in MN death. A study from the group of Christian M. Simon in this Frontiers Research Topic investigated p53-dependent nuclear upregulation of c-Fos protein in SMA mouse models, and suggested that nuclear c-Fos accumulation may serve as a novel marker for neuronal death in SMA. The necroptotic cell death pathway is another SMN-independent pathological mechanism which may have a role in SMA-associated motor neuron death. Chehade et al. in this Research Topic report on their studies with the receptor-interacting protein kinases (RIPK) RIPK1 and RIPK3, and Caspase-1 necroptosis pathways in the *Smn*^2*B*/−^ mouse model of SMA. The triple mutant (TKO) *Smn*^2*B*/−^*;Ripk3*^−/−^*;Casp1*^−/−^ displayed a robust increase in survival and improved motor function compared to *Smn*^2*B*/−^ mice. Larger muscle fibers were also observed in the TKO mice. The authors suggest the combination of small-molecule inhibitors of necroptosis and SMN-restoring drugs as a new strategy for SMA treatment. Recent studies suggest that post-translational modifications (PTMs) regulate the pleiotropic functions of SMN complex. In an article in this Research Topic, Riboldi et al. provide a thorough overview of the PTMs that are involved in the regulation of the SMN complex and SMA pathogenesis including phosphorylation, methylation, ubiquitination, acetylation, SUMOylating, and the crosstalk between PTMs of SMN.

The hallmark of SMA is the selective degeneration of alpha motor neurons in the brainstem and spinal cord, therefore it has been considered as a motor neuron-specific disease for decades. However, an increasing body of evidence suggest it involves many other physiological systems indicating it is necessary to consider SMA as a multi-system disorder (Lipnick et al., 2019; Yeo and Darras, 2020). This Research Topic was formally addressed authoritatively for the first time by scientists and clinicians from 11 different country on the 264th ENMC International workshop in Hoofddorp, the Netherlands, November 19–21st 2021. The workshop participants agreed that there is a multi-systemic pathology in SMA. They also summarized scientific evidence for multi-system defects including impaired myogenesis and synaptic development, spleen, immune system and liver involvement, vascularization defects and hypoxia, mitochondrial defects, collagen dysregulation, cardiac dysfunctions, cognitive impairment, metabolic abnormalities, and endocrine alterations (Detering et al., 2022).

Adding to above evidence, two articles in this Research Topic support the multi-systemic nature of SMA. Bonanno et al. evaluated the innate and adaptive immunity pattern in SMA patients before and after nusinersen treatment. They detected a significant increase in a spectrum of cytokines in serum of pediatric and adult SMA patients at baseline including IL-1b, IL-4, IL-6, IL-10, IFN-g, IL-17A, IL-22, IL-23, IL-31, and IL-33. After 6 months of nusinersen treatment, IL-4, IFN-g, Il-22, IL-23, and IL-33 significantly decreased in serum of pediatric SMA patients while IL-4, IL-6, INF-g, and IL-17A were significantly decreased in serum of adult SMA patients. The authors also reported the presence of inflammatory mediators in cerebrospinal fluid. Another article in this Frontiers Research Topic, Cui et al. reports on left ventricular strain imaging and the serum lipid profile related to cardiovascular disease in 80 later-onset SMA and 80 age-, gender-, and body surface area-matched control children. They reported lower measurement of global longitudinal strain and higher measurement of the time to peak longitudinal strain in SMA patients than control. They also reported higher level of total cholesterol, low-density lipoprotein (LDL)/HDL, and Apo B/Apo A1 levels in SMA patients. This data suggests the potential for SMA patients to be at increased risk for cardiomyopathy.

Recent approved SMN-restoring therapies (Sprinraza, Zolgensma, and Everysdi) have significant clinical impact in patients resulting in incremental improvements in motor function and developmental milestones, and preventing worsening of SMA symptoms. However, they are not curative. A significant number of patients respond poorly to these therapies, and their benefits vary among those that do respond (Day et al., 2022; Reilly et al., 2022). In this Frontiers Research Topic, Qiu et al. reviewed the development of nusinersen (the first SMA treatment), its contribution to the development of other drugs, and the potential of novel drug discovery with emerging strategies and new technology such as the clustered regularly interspaced short palindromic repeats (CRISPER) technology. Gene therapy for SMA represents a significant milestone in the treatment of neurologic disease. Zolgensma has demonstrated improved survival and motor milestone achievements for presymptomatic infants and patients with SMA1 (McMillan et al., 2022). A commentary article in this Research Topic from Rossoll and Singh discusses the challenges associated with SMA gene therapy including preparation, administration and the efficient body-wide distribution of the SMN1 gene delivered with the AAV9 vector. They also provide perspectives for the development of the next generation of gene therapies including alternative or additional routes of delivery, newer generation of AAV capsids, and optimized promoters and other tissue and cell-type specific control elements.

In summary, the insightful articles published in this Frontiers Research Topic provide new findings on the mechanisms of SMN pathology, evidence for multi-systematic defects in SMA, and perspectives for future treatment development. They also advocate for in-depth study of the systematic mechanisms of SMA pathology for identifying novel therapeutic targets for combinatorial approaches with SMN-restoring gene therapies.

## Author contributions

The author confirms being the sole contributor of this work and has approved it for publication.
